# Involvement of autophagy in the direct ER to vacuole protein trafficking route in plants

**DOI:** 10.3389/fpls.2014.00134

**Published:** 2014-04-08

**Authors:** Simon Michaeli, Tamar Avin-Wittenberg, Gad Galili

**Affiliations:** ^1^Department of Plant Sciences, The Weizmann Institute of ScienceRehovot, Israel; ^2^Max-Planck-Institut für Molekulare PflanzenphysiologiePotsdam-Golm, Germany

**Keywords:** plant autophagy, selective autophagy, plant vacuole, endoplasmic reticulum, seed storage proteins, golgi-independent trafficking, direct ER to vacuole, Atg8

## Abstract

Trafficking of proteins from the endoplasmic reticulum (ER) to the vacuole is a fundamental process in plants, being involved both in vacuole biogenesis as well as with plant growth and response to environmental stresses. Although the canonical transport of cellular components from the ER to the vacuole includes the Golgi apparatus as an intermediate compartment, there are multiple lines of evidence that support the existence of a direct ER-to-vacuole, Golgi-independent, trafficking route in plants that uses the autophagy machinery. Plant autophagy was initially described by electron microscopy, visualizing cellular structures that are morphologically reminiscent of autophagosomes. In some of these reports these structures were shown to transport vacuole residing proteins, particularly seed storage proteins, directly from the ER to the vacuole. More recently, following the discovery of the proteins of the core autophagy machinery, molecular tools were implemented in deciphering the involvement of autophagy in this special trafficking route. Here we review the relatively older and more recent scientific observations, supporting the involvement of autophagy in the special cellular trafficking pathways of plants.

## AUTOPHAGY AS AN ALTERNATIVE TRAFFICKING PATHWAY FOR PROTEINS DESTINED TO THE VACUOLE

Macroautophagy (termed hereafter simply autophagy) is a conserved cellular process that involves the sequestration of cytosolic components by a newly formed, double membrane vesicle termed autophagosome that is eventually directed to the cell’s lytic compartment (lysosome in animals or vacuole in plants and fungi; Li and Vierstra, 2012; Liu and Bassham, 2012). This process was originally thought to be a process of stress and starvation-induced bulk-degradation of cytosolic components. Autophagy may however, also act selectively, specifically targeting malfunctioning organelles such as mitochondria (mitophagy) or peroxisomes (pexophagy), protein aggregates (aggrephagy), invading pathogens (xenophagy), and even specific proteins (Floyd et al., 2012; Li and Vierstra, 2012). Notably, selective autophagy was also reported to act in special trafficking routs, delivering vacuolar resident proteins to function in this organelle. This role of autophagy in biogenesis-mediating process is in contrast to the degradative nature classically associated with it. Currently, the best known autophagy-dependent trafficking route is the cytosol to vacuole targeting (Cvt) pathway of yeast (Scott et al., 1996). This pathway involves the trafficking of at least two hydrolases, aminopeptidase 1 (Ape1), and α-manosidase (Ams1) from the cytosol into the vacuole. The Cvt pathway utilizes the core-autophagy machinery. The selectivity of this process is determined by Atg19 that attaches to the target proteins (Ape1 and Ams1) and can also bind Atg11 and Atg8 to mediate the formation of the Cvt vesicle (Lynch-Day and Klionsky, 2010). As will be further discussed here, analogous types of autophagy-dependent trafficking routes may well exist also in plants, especially for the direct route between the endoplasmic reticulum (ER), and the vacuole, bypassing the Golgi apparatus (De Marchis et al., 2013).

## AN AUTOPHAGY-RESEMBLING, DIRECT ER-TO-VACUOLE, GOLGI-BYPASSING TRAFFICKING ROUTE OF SEED STORAGE PROTEINS

**FIGURE 1:**
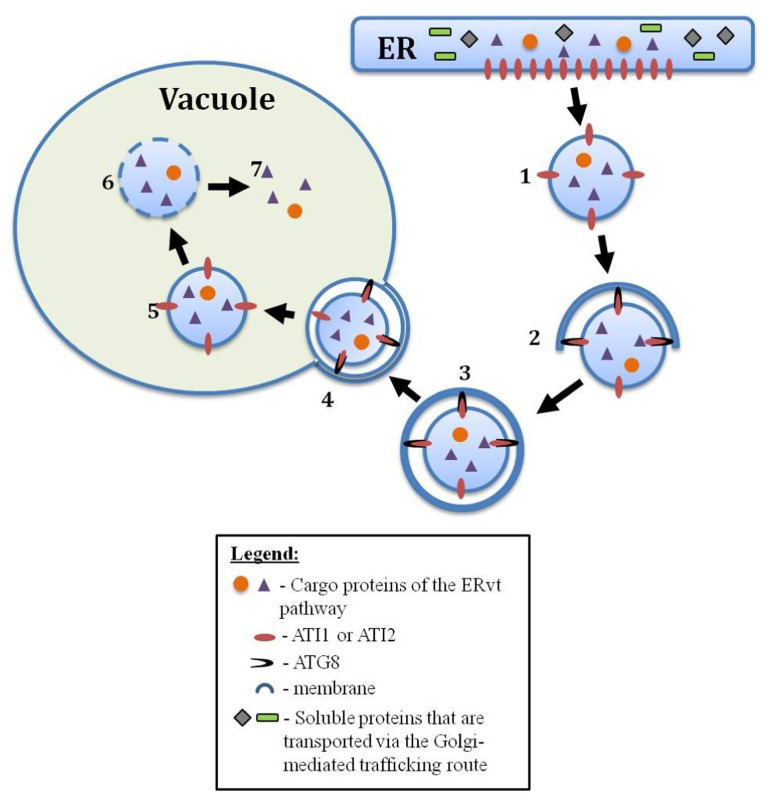
**A postulated model of the ERvt pathway in plants.** (1) An ERvt body, containing vacuole destined proteins (purple triangles/orange circles) such as storage proteins or proteases, buds from the ER. (2) with the aid of special cargo receptors (such as ATI1 and 2) that interact with Atg8, the ERvt body will be attached to an isolation membrane (phagophore), and (3) will be engulfed by this expanding membrane to form a special type of ERvt autophagosome. (4) This ERvt autophagosome will fuse with the tonoplast (5) releasing the ERvt body into the vacuole as an autophagic body (6) for its subsequent degradation, and (7) incorporation of the cargo proteins to the vacuole

Maturing plant seeds synthesize massive amounts of storage proteins whose mobilization during germination provides an important metabolic boost during early germination. The seed storage proteins of most plant species are synthesized within the ER and are then transported to protein storage vacuoles (PSVs) where they are packed in highly condensed forms (Herman and Larkins, 1999; Vitale and Hinz, 2005). Yet, in contrast to most other secretory proteins, the seed storage proteins are synthesized in massive amounts and also naturally possess an aggregative nature making them insoluble material that is not fit to be transported in “classical” trafficking routes (Herman, 2008). These facts question the ability and capacity of the classical ER-Golgi-vacuole route to account for the transport of the entire bulk of storage proteins to storage vacuoles. Indeed, extensive microscopic evidence suggest that seed storage proteins are transported from the ER to the storage vacuole also by an alternative route, a special ER-vacuole trafficking (ERvt) route that bypasses the Golgi (Galili, 2004; Herman and Schmidt, 2004). This ERvt route, originally discovered using electron microscopy observations of storage proteins in developing wheat seeds (Levanony et al., 1992; Galili et al., 1993), begins by the aggregation of the storage proteins within the ER, their subsequent budding from the ER to form special ER-derived PBs, and their internalization into the storage vacuoles by a process that strongly resembles autophagy (Hara-Nishimura et al., 1998; Robinson et al., 2005; Herman, 2008; Ibl and Stoger, 2011; Wang et al., 2011). Protein aggregation in the ER may also be the fate of non-storage proteins following stresses that hamper proper protein folding (a phenomenon known as ER-stress) resulting in the unfolded protein response (UPR; Howell, 2013). Recently it was shown that inducing ER stress in *Arabidopsis* results in the delivery of ER components, such as ER membrane decorated with ribosomes, to vacuoles via autophagy (Liu et al., 2012), further strengthening the possible involvement of autophagy in storage protein trafficking. Naturally, the ERvt autophagy is distinct from the classical non-selective starvation-induced autophagy by being selective to transport only storage proteins (**Figure 1**). Whether these two autophagy processes use the same or different machineries is an interesting issue that will be addressed further ahead in this review. The ERvt autophagy route appears to be dependent on the type of the storage proteins as well as the plant species and tissues where they are synthesized. The storage proteins of maize seeds accumulate entirely within ER-derived PBs. Surprisingly, expression of these storage proteins genes in transgenic tobacco plants resulted in their transport to the vacuole by autophagy (Hoffman et al., 1987). An analogous Golgi-bypass route was further proposed for: (i) the transport of specific precursors of storage proteins from the ER to the vacuole via “precursor-accumulating” (PAC) bodies (Hara-Nishimura et al., 1998); (ii) the transport of germination-associated proteases, containing a C-terminal K/HDEL ER-retention signal, from the ER to the vacuole (this will be elaborated on in the next section); and (iii) the transport of storage proteins to the storage vacuole in aleurone cells of developing maize seeds (Reyes et al., 2011).

Reyes et al. (2011) presented data from *in vivo* imaging and electron tomography, suggesting that in maize seed aleurone cells, storage proteins are transported from the ER to PSVs using a special autophagic process. This process also appears to be similar to the ERvt process described in wheat (Levanony et al., 1992; Galili et al., 1993; Ibl and Stoger, 2011). Classically, the selection of cargo to be delivered by the autophagosome is mediated by binding of Atg8, residing on the growing autophagosome membrane, to the cargo protein or an adaptor protein linking the cargo to Atg8 (Li and Vierstra, 2012). Despite microscopic evidence suggesting the involvement of autophagy in storage protein transport, no co-localization of the maize seed storage proteins with Atg8 was observed in steady state microscopy images used in this report (Reyes et al., 2011). Reyes and associates claimed that the lack of co-localization might stem from the function of a non-canonical autophagy mechanism, not involving Atg8. Other, non-canonical (alternative) autophagic pathways may exist, as an *Atg5*/*Atg7* independent autophagy was demonstrated in mammalian embryonic tissues (Nishida et al., 2009). However, another possibility could be a transient interaction between Atg8 and the potential cargo receptor proteins that might be localized on the surface of storage protein bodies. This may result in low co-localization frequency, which was possibly very difficult to detect by steady-state microscopic analysis. A similar observation was recently made in our group regarding the Atg8 interacting proteins, ATI1 and ATI2. *In vivo* interaction of Atg8f with either of these two proteins was detected, but co-localization was quite infrequent (Honig et al., 2012). Also, it is important to bear in mind that autophagy components are involved, not only in autophagosome biogenesis, but also in its transport to the vacuole, its fusion with the tonoplast and in the delivery of the autophagic-body into the vacuole lumen. Thus, evaluating the involvement of autophagy in any process must include each of these phases, usually by looking whether they are hampered in the background of autophagy deficient mutants (Klionsky et al., 2012).

## ERvt PATHWAYS TRANSPORTING GERMINATION AND DEFENSE-ASSOCIATED CYSTEINE PROTEASES TO THE VACUOLE

An ERvt route appears to be involved not only in the deposition of seed storage proteins inside PSVs during seed maturation, but also in their mobilization during early germination. Mobilization of the storage proteins during germination depends on the *de novo* synthesis of a family of cysteine proteases, which are inserted into the ER and are then transported to PSVs. Intriguingly, a number of these cysteine proteases contain a C-terminal K/HDEL signal, which classically functions in the retention of ER-resident proteins within this organelle (Toyooka et al., 2000). Moreover, the K/HDEL signal appears to be essential for the trafficking of these cysteine proteases to the PSV because removal of this signal leads to their secretion (Okamoto et al., 2003). The nascent K/HDEL-containing cysteine proteases are transiently retained within the ER, enabling their deposition into special vesicles, called KDEL vesicles (KV), which bud directly from the ER and are then internalized into the vacuole (Toyooka et al., 2000). Notably, when expressed in the heterogonous system of insect Sf-9 cells, the KDEL ER-retention sequence is post-translationally removed from this protein either within the ER or in a related compartment (Okomoto et al., 1999), implying that a similar mechanism of enzymatic removal of this ER-retention signal may also exist in plants and may be important for the transport of this protein to the vacuole.

Different cysteine proteases lacking a KDEL ER retention signal accumulate in a different organelle, namely, the spindle-shaped ER-body of *Arabidopsis* that is considered to be the largest ER-derived body in plants (Yamada et al., 2011). These cysteine proteases are the stress induced RD21 and γ-vacuolar processing enzyme (VPE) proteins. ER-bodies were seen fusing with the tonoplast following salt treatment mediating the delivery of RD21 and γ-VPE to the vacuole (Hayashi et al., 2001), thus defining another unique mode of ERvt. ER bodies are increasingly associated with defense against plant pests and fungi (Yamada et al., 2011). This illustrates that mobilization of proteins from the ER to the vacuole via ER bodies might be important for both biotic and abiotic stress tolerance. Notably, thus far no direct evidence linking autophagy with either KV or ER-body pathways have been reported.

## SELECTIVE-AUTOPHAGY FACILITATING A STRESS-INDUCED, Atg8-MEDIATED, DIRECT ER-TO-VACUOLE TRAFFICKING

Although the issue of the identity of the membrane donor for autophagosome biogenesis is controversial (Rubinsztein et al., 2012), the ER is considered one of the prominent candidates for this process (Hayashi-Nishino et al., 2009). Also, it is now recognized that following ER-stress, ER components are delivered for degradation via autophagy in both yeast and mammalian cells (Bernales et al., 2006; Ogata et al., 2006; Deegan et al., 2013). In this respect, plants are not different from other eukaryotes. Recently, the delivery of ER components, including ER-membrane decorated with ribosomes, was demonstrated to occur in plants cells following ER-stress (Liu et al., 2012). In this report, the accumulation of Atg8-positive bodies that co-localize with the ER marker, GFP-HDEL, was detected following ER-stress. Also, the presence of autophagic-bodies containing ER membrane was detected by electron-microscopy in vacuoles of ER-stressed plants. Finally, IRE1b, a special ER-stress sensor, was shown to be required for this autophagic process (Liu et al., 2012). Nevertheless, this most probably reflects the degradation of ER components and not the delivery of vacuole residing proteins that function in this organelle.

A support for the involvement of autophagy in the transport of functional cargo from the ER to the vacuole has recently been obtained, studying two novel, closely related, *Arabidopsis* proteins, termed Autophagy Interacting proteins one and two (ATI1 and ATI2; Honig et al., 2012). Each of these proteins was shown to contain two consensus Atg8-binding motifs and also bind the *Arabidopsis* autophagy-associated Atg8f protein based on both the yeast 2-hybrid as well as the *in vivo* bimolecular fluorescence complementation (BiFC) approaches. Under normal growth conditions both ATI1 and ATI2 were localized to the ER. However, upon exposure to carbon starvation, the two proteins were incorporated into newly identified bodies that are not “classical” autophagosomes and are moving dynamically along the ER network. Subsequently, these ATI1 and two labeled bodies (ATI-bodies) are transported from the ER to the vacuole (Honig et al., 2012). These results provided the first hard-core evidence for the involvement of autophagy in the transport of cargo from the ER to the vacuole in plants. It also demonstrated that alternative pathways that involve autophagic components but are not “classical” autophagy do exist in plants. It was thus suggested that the ATI1 and ATI2 mediated transport from the ER might represent a pathway homologous to yeast Cvt, in that it mediates the transport of functional proteins to the vacuole, similarly to the route occurring in wheat (Li and Vierstra, 2012). A general model, depicting an ERvt body that originates from the ER and is delivered to the vacuole through the interaction of special cargo-receptors (such as ATI1 and ATI2) with Atg8, is presented in **Figure 1**.

The ability of autophagy to interact with components from other trafficking pathways and to deliver a variety of substrates directly to the vacuole was also recently demonstrated by the discovery of anthocyanin containing bodies that are labeled by both Atg8 and Exo70B1, a subunit of an exocyst complex (Kulich et al., 2013). Exocyst complexes are classically involved in the tethering of exocytosis-related vesicles to the plasma membrane, thus mediating their initial contact (Synek et al., 2014). Interestingly, significantly less mRFP-Atg8f positive bodies were found within vacuoles of Exo70B1 deficient plants compared to WT plants, implying for a direct role of Exo70B1 in the trafficking of anthocyanin autophagosomes to the vacuole (Kulich et al., 2013). Finally, it is important to mention also non-storage, lytic-vacuole residing proteins that were shown to reach the vacuole in a Golgi-independent manner. These are members of the tonoplast intrinsic protein (TIP) family (Gomez and Chrispeels, 1993; Rivera-Serrano et al., 2012) and vacuolar H-driven ATPase subunits (VHA; Schumacher and Krebs, 2010). Although autophagy was not shown to be involved in TIPs trafficking, VHA-a subunits were recently utilized, among other markers, to monitor vacuole biogenesis in root meristematic cells, where autophagosome-like structures have been observed (Viotti et al., 2013) as will be discussed in the next section.

## CONTRIBUTION OF MATERIAL FROM THE ER TO THE BIOGENESIS OF THE LYTIC VACUOLE OCCURS THROUGH A GOLGI-BYPASSING TRAFFICKING ROUTE

Autophagy was previously suggested to be involved in plant vacuole biogenesis (Marty, 1999) and it was also suggested that this is not the “classically” starvation induced autophagy, but rather a unique type of autophagy dedicated to the process of vacuole biogenesis (Yano et al., 2007). In animals, contribution of autophagy to replenish lysosome pool following starvation was reported in rat kidney cells. This process, termed autophagic lysosome reformation (ALR), seems to be conserved in multiple animal species (Yu et al., 2010). Furthermore, the *Arabidopsis* deubiquitinating enzyme, AMSH1, a close homolog of AMSH3 that is essential for proper vacuole biogenesis (Isono et al., 2010), was shown to be necessary for proper autophagy (Katsiarimpa et al., 2013).

Recently, Viotti et al. (2013) demonstrated how the ER is the main membrane source for vacuole biogenesis in root meristematic cells. This contribution of membrane, sterol and specific protein pumps (vacuolar H^+^-PPase and the vacuolar H^+^-ATPase) occurs by a Golgi-bypassing trafficking route that seems homologous to ERvt described earlier. Also here, provacuoles that are considered precursors of the lytic vacuole displayed autophagosome-like structure of double or multilayered membranes (Viotti et al., 2013). Nonetheless, similarly to the report of storage proteins in maize aleurone cells (Reyes et al., 2011), ER-derived provacuoles were not labeled by Atg8 markers and their accumulation was not affected by *Atg2*, *Atg5,* or *Atg7* knockouts (Viotti et al., 2013). Thus, autophagy is probably not involved in the biogenesis of these provacuoles. Yet, it is possible that Atg8 transiently or rarely interacts with these provacuoles (as was demonstrated for ATI- bodies). Also, as already discussed, it is essential to test whether the delivery of cargo to vacuoles is occurring in *Atg* mutants before conclusions can be drawn regarding the involvement of autophagy (Klionsky et al., 2012). Though the authors showed the presence of provacuoles in *Atg* mutants, data regarding their trafficking to *Atg* mutant vacuoles was not presented (Viotti et al., 2013). Thus, currently it is not clear whether core-autophagy proteins are involved in provacuoles trafficking and vacuole biogenesis.

## CONCLUSION

There is no doubt that the trafficking pathways described here occur in plants and are distinct from the known Cvt pathway of yeast. The Cvt pathway delivers cargo from the cytoplasm to the vacuole while the plant pathways direct proteins from the ER to the vacuole. In addition, although the core-autophagy machinery is essential for the biogenesis of the Cvt vesicles, it does not seem to be essential for the biogenesis of the plant ERvt vesicles in plants. Nevertheless, the morphological resemblance of ERvt vesicles to autophagosomes, coupled with the involvement of Atg8 in such processes as described for the ATI-bodies, strongly suggests the involvement of autophagy in ERvt pathways in plants. Most probably this involvement is taking place in the transport of the ERvt vesicles to the vacuole following their autophagy-independent biogenesis (**Figure 1**). Yet, this hypothesis awaits clear evidences that can be supplied by further investigating both ATI-bodies in *Arabidopsis* and storage proteins trafficking in cereal seeds. This can be examined by evaluating whether these types of ERvt vesicles reach the vacuole in the background of classical autophagy deficient plants such as *Atg5*, *Atg7,* and *Atg4a4b*.

## Conflict of Interest Statement

The authors declare that the research was conducted in the absence of any commercial or financial relationships that could be construed as a potential conflict of interest.
